# Trabectedin Followed by Irinotecan Can Stabilize Disease in Advanced Translocation-Positive Sarcomas with Acceptable Toxicity

**DOI:** 10.1155/2016/7461783

**Published:** 2016-10-24

**Authors:** J. Herzog, F. von Klot-Heydenfeldt, S. Jabar, A. Ranft, C. Rossig, U. Dirksen, J. Van den Brande, M. D'Incalci, I. von Luettichau, P. J. Grohar, W. E. Berdel, St. Burdach

**Affiliations:** ^1^Division of Pediatric Hematology/Oncology, Department of Pediatrics, Comprehensive Cancer Center Munich, Kinderklinik München Schwabing, Klinikum Rechts der Isar, Technische Universität München, München, Germany; ^2^Department of Pediatric Hematology and Oncology, University Children's Hospital Muenster, Muenster, Germany; ^3^University of Antwerp, Antwerp University Hospital, Wilrijkstraat 10, 2650 Edegem, Belgium; ^4^Department of Oncology, Laboratory of Cancer Pharmacology, IRCCS-Istituto di Ricerche Farmacologiche Mario Negri, Milan, Italy; ^5^Department of Pediatrics, Van Andel Institute, Helen DeVos Children's Hospital and Michigan State University, Grand Rapids, MI, USA; ^6^Department of Medicine A, Hematology and Oncology, University of Muenster, Muenster, Germany

## Abstract

*Background*. Preclinical data indicate that trabectedin followed by irinotecan has strong synergistic effects on Ewing sarcoma. This is presumably due to hypersensitization of the tumor cells to the camptothecin as an effect of trabectedin in addition to synergistic suppression of EWS-FLI1 downstream targets. A strong effect was also reported in a human rhabdomyosarcoma xenograft.* Procedure*. Twelve patients with end-stage refractory translocation-positive sarcomas were treated with trabectedin followed by irinotecan within a compassionate use program. Eight patients had Ewing sarcoma and four patients had other translocation-positive sarcomas.* Results*. Three-month survival rate was 0.75 after the start of this therapy. One patient achieved a partial response according to RECIST criteria, five had stable disease, and the remaining six progressed through therapy. The majority of patients experienced significant hematological toxicity (grades 3 and 4). Reversible liver toxicity and diarrhea also occurred.* Conclusions*. Our experience with the combination of trabectedin followed with irinotecan in patients with advanced sarcomas showed promising results in controlling refractory solid tumors. While the hematological toxicity was significant, it was reversible. Quality of life during therapy was maintained. These observations encourage a larger clinical trial.

## 1. Background

Trabectedin, a marine alkaloid, is a minor groove-binding agent that blocks the cell cycle in late S- and G-phase, affects gene transcription, and impairs DNA-repair [1]. The drug has been shown to induce a variety of effects. While it causes cell cycle arrest and cell death in the cancer cell itself, it also has immunomodulatory activity [2]. By inhibition of proinflammatory and angiogenic cytokines trabectedin changes the tumor microenvironment [3]. Irinotecan, a camptothecin prodrug, which is converted to SN-38, a topoisomerase I inhibitor, induces cell death in S-phase. While it shows modest single agent activity [4, 5], better results have been achieved in combination with other cytotoxic drugs [6, 7]. Grohar et al. recently reported preclinical data of a Ewing sarcoma xenograft model, suggesting a strong synergism of trabectedin and irinotecan given sequentially [8]. Trabectedin blocks EWS-FLI1 activity, leading to suppression of Werner's syndrome gene (WRN). WRN deficient cells are known to be hypersensitive to camptothecins; hence trabectedin sensitizes specifically the Ewing tumor cells to treatment with irinotecan. In addition, irinotecan augments the trabectedin mediated suppression of EWS-FLI1 activity and downstream target expression [9]. A similar striking synergism between the two drugs was observed in a human rhabdomyosarcoma xenograft model by Riccardi et al. [10], though the underlying mechanism in this setting is unclear.

Based on these promising preclinical results twelve patients with refractory sarcomas with no established treatment options were treated with a regimen combining trabectedin and irinotecan.

## 2. Materials and Methods

### 2.1. Patients

Between October 2013 and December 2014 ten patients with translocation-positive pediatric-type refractory and end-stage sarcomas with no conventional treatment options left started treatment at our institutions with an off-label compassionate use chemotherapy, combining trabectedin and irinotecan based on preclinical observations. Two additional patients, one in Italy and one in Belgium, underwent a similar regimen and are included in this report. All patients were evaluable for toxicity and response. Patient characteristics are presented in Table 1. Eight patients had Ewing sarcoma. Four had other translocation-positive soft tissue sarcomas: two alveolar rhabdomyosarcomas, one synovial sarcoma, and one desmoplastic small round cell tumor. Patients were between 6 and 57 years of age at diagnosis (median 18 years) and between 12 and 60 years of age at initiation of therapy (median 26 years). They were 33% female and 67% male (Table 1). All patients showed metastatic, progressive disease at initiation of therapy with a median history of 4 years of cytotoxic cancer therapy. All patients were pretreated with chemotherapy according to standard protocols (CWS, EURO-E.W.I.N.G. or comparable regimen) and had received at least one second-line therapy. Six patients were previously treated with irinotecan until progression and assumed to be refractory to this drug without the addition of trabectedin (patient nos. (6), (7), (9), (10), (11), and (12)). Informed consent was obtained from the patients and/or their parental guardians, depending on the age of the patients. Publication of the data is in accordance with § 37 of the Declaration of Helsinki (64th WMA General Assembly, Fortaleza, Brazil, Oct 2013).

### 2.2. Treatment Regimen

**Figure 1:**
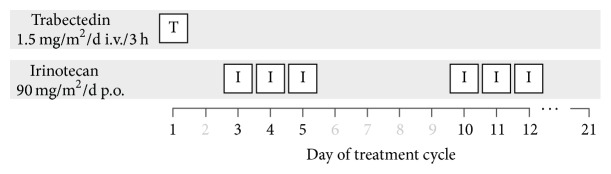
Treatment plan. Duration of cycle: 21 days. Response evaluation: after 2 cycles.

In a treatment course of 21 days trabectedin was administered i.v. at a maximum dose of 1.5 mg/m^2^ on day 1, followed by irinotecan p.o. on days 3–5 and 10–12 at a maximum dose of 90 mg/m^2^ (Figure 1). A total of 47 courses were administered. Trabectedin was given as a 3- or 24-hour infusion. Irinotecan, if possible, was taken p.o. by the patient and dose adjusted depending on the patient's pretreatment. Alternatively patients, who did not tolerate oral irinotecan, received the drug intravenously at a maximum dose of 90 mg/m^2^. The majority of patients received steroid pretreatment to ameliorate liver toxicity depending on the institutions guidelines, as recommended by the manufacturer. Two patients underwent a slightly different treatment schedule: one patient (no. (5)) received trabectedin at a dose of 1 mg/m^2^ i.v./24 h on day 1, irinotecan 75 mg/m^2^ i.v. on day 2 for six cycles, and irinotecan on days 2 and 4 for five subsequent cycles, with treatment cycles of four weeks. The other patient (no. (6)) was scheduled to receive trabectedin at 1.1 mg/m^2^ on day 1 and irinotecan 80 mg/m^2^ on days 2–4 of a 21-day cycle. Due to serious hematological, gastrointestinal toxicity and infectious complications, irinotecan was skipped for the 2nd cycle. In the following cycle, the patient received 10% of the initial irinotecan dose, but again dose limiting toxicity was observed. As a result, trabectedin was continued as single agent, and the patient was censored at that point.

Baseline functional imaging (PET-MRI or PET-CT) including definition and measurement of index lesions were performed before the first course and after every two treatment cycles. Toxicity was graded according to NCI-CTCAE V4. Patients were monitored with blood counts, liver function tests, and clinical examination at least twice a week depending on laboratory values and clinical performance. Subsequent courses were started at day 22 or after recovery of drug induced toxicity to baseline or grade 1. Measurable tumor response was evaluated according to RECIST.

### 2.3. Retrospective Statistics

**Figure 2:**
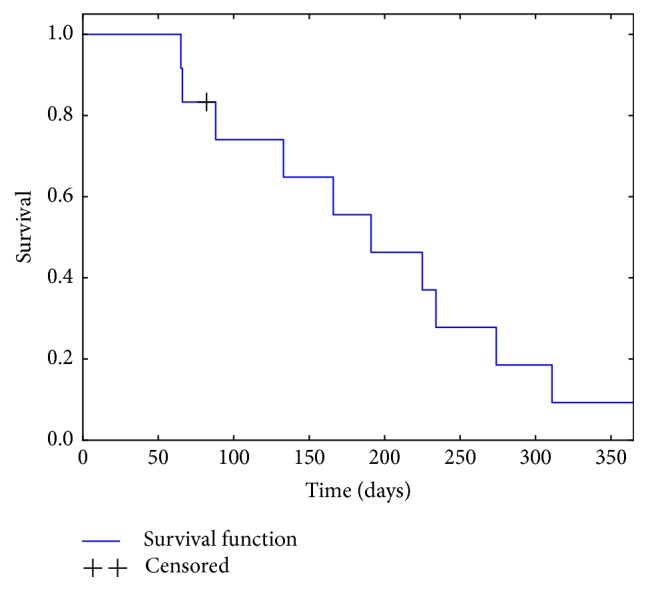
Cumulative survival (2/12, Kaplan-Meier estimate).

For survival analysis the Kaplan-Meier method was used (Figure 2). For a matched pair analysis for the patients with Ewing sarcoma blinded retrospective data from the EURO-E.W.I.N.G. 99/EWING 2008 trial was obtained. Matching criteria were age, sex, metastasized primary disease, time to first relapse, number of relapses, and type of relapse (localized/systemic/combined). 6/8 patients could be matched successfully.

## 3. Results

### 3.1. Toxicity

In the majority of patients, the tolerability of the combination of trabectedin and irinotecan was acceptable. All patients experienced grade 3 or grade 4 hematological toxicity with neutropenia being the most common toxicity, but thrombocytopenia and anemia occurring as well. Reversible liver toxicity (grades 3 and 4) was seen in seven patients, and diarrhea (grades 3 and 4) was also observed in three patients. Only one patient suffered from dose limiting diarrhea and severe prolonged neutropenia, so that irinotecan had to be reduced and finally omitted. One patient suffered from grade 3 pancreatitis after the first cycle of treatment. 73% of courses of trabectedin were administered with premedication with dexamethasone or methylprednisolone according to the manufacturer's recommendation. We did not see a significant difference in hematological or liver toxicity after premedication with dexamethasone.

### 3.2. Response

Six patients achieved stable disease at the first evaluation after two courses of therapy while six patients progressed on therapy. As of August 2016 1/6 of the patients who showed stable disease after two cycles was still alive (no. (6), censored after 3 cycles, continued with trabectedin as a single agent). The patient with a partial response after five courses (no. (3)) (Figure 4) received a total of 7 cycles, before he progressed under therapy and died of disease 17.3 months after starting therapy. Two patients (nos. (8) and (9)) showed progressive disease after 3 cycles, left the protocol, and died of disease 7.8 months (no. (8)) and 5.6 months (no. (9)) after starting therapy. One of the six patients with stable disease after one cycle (no. (1)) according to imaging criteria did not continue therapy due to his poor overall condition and died of disease progression two months later. The last one of these six patients (no. (5)) was evaluated after three cycles and also showed stable disease. He received additionally eight more cycles and died presumably of a chemotherapy unrelated cerebral stroke 304 days after starting therapy, as published in a separate case report by Tancredi et al. [11].

The overall survival was 8.4 months (2.2 to 25.8 months) including all twelve patients; median survival was 6.4 months. Overall survival in the patients who achieved at least stable disease was 11.7 months (3.0 to 25.8 months); progression-free survival was 6.7 months (1.0 to 13.6 months) for this group. For the patients who did not respond to treatment overall survival was 5.3 months (2.2 to 9.2 months).

**Figure 3:**
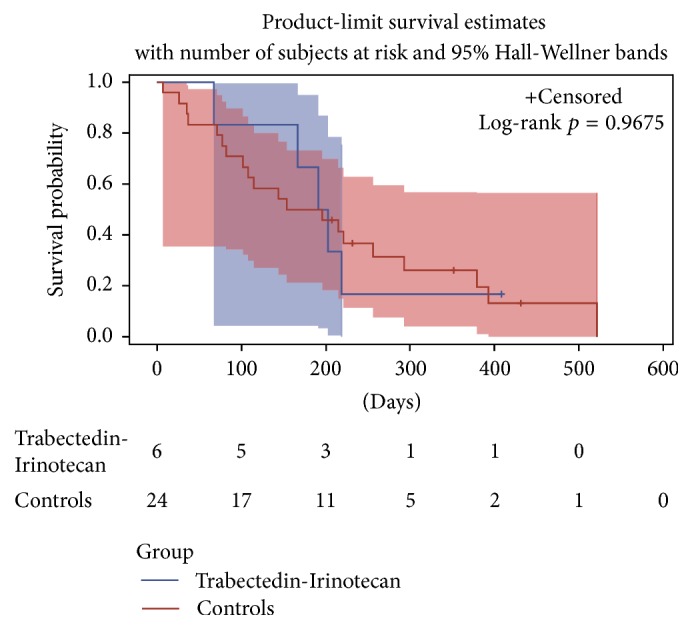
Matched pair analysis Ewing sarcoma patients.

**Figure 4:**
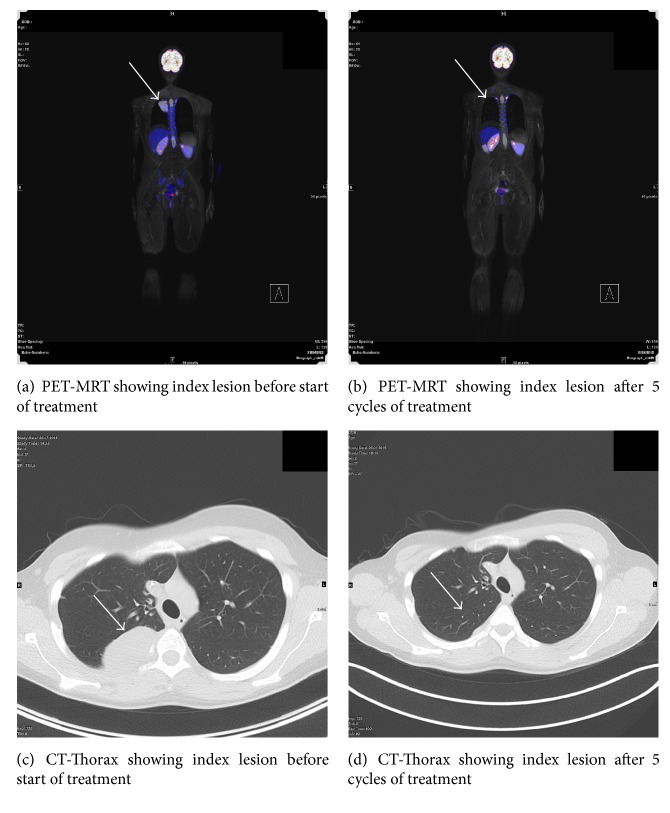
Tumor response after 5 cycles of trabectedin/irinotecan, patient with synovial sarcoma.

Finally of the 8 patients with Ewing sarcoma enrolled on this trial, one was censored and 4 of the remaining 7 initially responded with stable disease or better. The matched pair analysis for patients with Ewing sarcoma including 6/8 patients (Figure 3) did not show a significant difference in survival between the trabectedin-irinotecan group and the comparison group (*p* = 0.976), though median survival was longer with 6.6 months (2.2 to 13.6 months) in the irinotecan-trabectedin group versus 5.8 months (0.2 to 17.4 months) (*p* = 0.808) in the comparison group.

## 4. Discussion

Considering the special characteristics of the reported patients, progressive, metastatic, and refractory disease, the treatment was well tolerated. Stable disease in six out of twelve evaluable patients with one of those later achieving a partial remission presents a promising treatment option for refractory pediatric-type sarcomas. While toxicity is significant, it is manageable and completely reversible. Even in patients who had previously progressed during irinotecan, the combined treatment of trabectedin and irinotecan was able to overcome treatment resistance in 3/6 patients and lead to stable disease. The matched pair analysis confirms that the evaluated regimen is at least equivalent to other regimens for Ewing sarcoma patients. The authors are of course aware of the limitations of this retrospective evaluation of a case series. It must be emphasized that due to the heterogeneity of tumor entities, different pretreatments, and wide range of age and adjustments in trabectedin and irinotecan dose only limited conclusions about the general efficacy of the regimen can be made. Thus, a prospective clinical study to investigate the potential of sequential treatment with trabectedin and irinotecan, including possible identification of biomarkers for sensitivity versus resistance, is warranted.

## Figures and Tables

**Table 1:** Patient characteristics.

Patient	Diagnosis (fusion protein/cytogenetic finding)	Age	Age at FD^(a)^	Sex	Number of prior chemoregimens	Prior radiation therapy	Total number of cycles received	Maximum toxicities	Best response
(1)	Ewing sarcoma (second malignancy, initially fibrosarcoma)^(b)^	21	4	m	7	Yes	1	Leukopenia G3	SD^(c)^, DOD^(d)^
(2)	Alveolar rhabdomyosarcoma (PAX3-FKHR/t(2;13)(q35;q14))	17	13	m	3	Yes	2	Leukopenia G4, thrombocytopenia G4, hepatic G4, pancreatitis G3, infection G3	PD^(e)^, DOD^(d)^
(3)	Synovial sarcoma (SYT-SSX2/t(X;18))	18	8	m	2	No	7	Pancytopenia G4, hepatic G4, infection G3	PR^(g)^, DOD^(d)^
(4)	Ewing sarcoma (EWS-FLI1/t(11;22)(q24;q12))	18	13	f	2	Yes	2	Hepatic G3	PD^(e)^, DOD^(d)^
(5)	Ewing sarcoma (EWS-FLI1/t(11;22)(q24;q12))	43	11	m	3	Yes	11	Hepatic G3	SD^(c)^, DOOC^(g)^
(6)	Ewing sarcoma (EWS-FLI1/t(11;22)(q24;q12))	28	22	m	2^(h)^	Yes	3^+^	Diarrhea G4, pancytopenia G4, infection G4	SD^(c)^
(7)	Ewing sarcoma (EWSR1-FLI1/t(11;22)(q24;q12); type II (EWSR1-Exon7/FLI1-Exon5))	35	16	m	6^(h)^	Yes	2	Leukopenia G4, mucositis G3, hepatic G3	PD^(e)^, DOD^(d)^
(8)	Ewing sarcoma (EWSR1-FLI1/t(11;22)(q24;q12))	60	57	f	5	Yes	3	Pancytopenia G4, FUO in Neutropenia G4	SD^(c)^, DOD^(d)^
(9)	Ewing sarcoma (EWSR1-FLI1/t(11;22)(q24;q12))	24	20	f	6^(h)^	Yes	3	FUO in neutropenia G4, mucositis G3, diarrhea G3	SD^(c)^, DOD^(d)^
(10)	Ewing sarcoma (EWSR1-FLI1/t(11;22)(q24;q1); EWSR1-Exon 7/FLI1-Exon 6; type I))	24	14	m	6^(h)^	Yes	2	No toxicity	PD^(e)^, DOD^(d)^
(11)	Desmoplastic small round cell tumor (EWSR1-WT1/t(11;22)(p13;q12))	12	6	m	3^(h)^	Yes	6	Pancytopenia G4, hepatic G4	PD^(e)^, DOD^(d)^
(12)	Alveolar rhabdomyosarcoma (PAX3-FKHR/t(2;13)(q35;q14))	17	15	f	2^(h)^	Yes	5	Pancytopenia G4, hepatic G3	PD^(e)^, DOD^(d)^

(a) FD: first diagnosis; (b) while histological diagnosis of the patient's second malignancy was Ewing sarcoma, the EWSR1-FLI1 translocation could neither be confirmed nor ruled out conclusively; (c) SD: stable disease; (d) DOD: death of disease; (e) PD: progressive disease; (f) PR: partial remission (continued therapy until PD and DOD); (g) DOOC: death of other causes; (h) prior exposure to irinotecan. (+) indicates that the patient is still on therapy; Pt. no. 6 continued with trabectedin as a single agent.
